# Manifestations neuropsychiatriques au cours du lupus érythémateux systémique: à propos de 108 cas au Cameroun

**DOI:** 10.11604/pamj.2022.42.241.27227

**Published:** 2022-07-28

**Authors:** Mapoure Njankouo Yacouba, Same Bebey Francine, Balla Nde Jean Francois, Eloundou Onomo Paul, Kenmegne Caroline, Gams Massi Daniel, Doumbe Jacques, Ngandeu Singwe Madeleine

**Affiliations:** 1 Department of Clinical Sciences, University of Douala, Douala, Cameroon,; 2 Douala General Hospital, Neurological Unit, Douala, Cameroon,; 3 Douala Laquintinie Hospital, Douala, Cameroon,; 4 Cité Verte District Hospital, Yaoundé, Cameroon,; 5 Department of Clinical Sciences, University of Yaoundé I, Yaoundé, Cameroon,; 6 Yaoundé Central Hospital, Yaoundé, Cameroon

**Keywords:** Neurolupus, lupus érythémateux systémique (LES), Cameroun, Neurolupus, systemic lupus erythematosus (SLE), Cameroon

## Abstract

Peu de données sur les atteintes neuropsychiatriques au cours du lupus érythémateux systémique (NPSLE) sont disponibles en Afrique subsaharienne. Le but de cette étude était de déterminer leur fréquence et d´en décrire les caractéristiques au Cameroun. Il s´agit d´une étude rétrospective menée dans 3 hôpitaux du Cameroun colligeant tous les dossiers de patients lupiques de l´unité de rhumatologie de 2009 à 2019. L´évaluation de l´activité lupique était réalisée à l´aide du systemic lupus erythematosus activity index (SLEDAI). Au total, 108 dossiers de patients d´âge moyen de 40,2 ± 13,7 ans étaient inclus. La fréquence du NPSLE était de 55,5% (n = 60). Les NPSLE étaient présentes au diagnostic initial du LES chez 37,0% (n = 40) et survenaient chez 20 patients (18,5%) au cours de la première année. Lorsque le NPSLE était inaugural, l´atteinte du système nerveux central était dominante avec le syndrome démyélinisant 27,8% (n=30) et les céphalées 21,3% (n=23). La mononeuropathie était l´atteinte du système nerveux périphérique la plus importante soit 15,7% (n=17). Les facteurs associés à la survenue des NPSLE étaient le rash malaire (p=0,024), l´alopécie (p=0,024), l´activité lupique très élevée (p=0,011), l´arthralgie (p<0,001), le facteur anti-nucléaire (p=0,002). La présence des NPSLE ne semblait influencer ni l´activité lupique (log rank p=0,227), ni la probabilité de survenue d´une nouvelle poussée lupique (log rank p=0,233). Plus de la moitié de nos patients présentait un NPSLE au cours de la première année. La présence de signes cutanés et articulaires, d´une activité lupique élevée, et de facteur anti-nucléaire étaient associés à la survenue de NPSLE.

## Abstract (en)

Few data on neuropsychiatric disorders in systemic lupus erythematosus (NPSLE) are available in sub-Saharan Africa. The purpose of this study was to determine their frequency and describe their features in Cameroon. We conducted a retrospective study collecting all the medical records of patients with systemic lupus erythematosus (SLE) hospitalized in the department of rheumatology of 3 hospitals in Cameroon from 2009 to 2019. Lupus activity was assessed using the SLE activity index (SLEDAI). A total of 108 records of patients with a mean age of 40.2 ± 13.7 years were included in the study. The frequency of NPSLE was 55.5% (n=60). Neuropsychiatric disorders were diagnosed concomitantly with SLE (37.0%; n = 40) while in 20 patients with SLE (18.5%) they occurred during the first year. When NPSLE were inaugural, central nervous system involvement was dominant, with demyelinating syndrome 27.8% (n=30) and headaches 21.3% (n=23). Mononeuropathy was the most frequent peripheral nervous system involvement (15.7%; n=17). Factors associated with NPSLE occurrence were malar rash (p=0.024), alopecia (p=0.024), very high lupus activity (p=0.011), arthralgia (p<0.001), anti-nuclear factor (p=0.002). NPSLE did not appear to influence either lupus activity (log rank p=0.227) or the probability of new onset lupus (log rank p=0.233). More than half of patients had NPSLE during the first year. The presence of cutaneous and articular signs, high lupus activity, and anti-nuclear factor were associated with the occurrence of NPSLE.

## Introduction

Le lupus érythémateux systémique (LES), est une maladie auto-immune non spécifique d´organe caractérisée par un polymorphisme clinique et représente l´archétype des maladies auto-immunes systémiques [1,2]. Il est ubiquitaire, rencontrée fréquemment chez les femmes noires en âge de procréer. Sa prévalence mondiale est de 20-150 cas pour 100000 habitants avec une incidence de 1 à 25 cas pour 100000 habitants [2].

L´atteinte neuropsychiatrique au cours du LES multiplie par dix le risque de mortalité par rapport à la population générale [3]. *L´American College of Rheumatology (ACR)* publie en 1999 une classification des *NPSLE* incluant douze atteintes neurologiques centrales parmi lesquelles quatre atteintes psychiatriques et sept atteintes périphériques [4]. Le *NPLSE* peut être inaugural comme le rapporte l´étude de cohorte menée par Hanly *et al*. dans 39-50% des cas [5-8]. Bien que certaines études de cohorte telles que Lumina et celle de Maryland cohorte aient décrit des cas sévères de *NPSLE* chez les patients caucasiens, l´atteinte neuropsychiatrique est fréquente chez les Afro-Américains, les hispaniques et les asiatiques [6-10]. En Asie, des études portant sur la qualité de vie des malades lupiques ont montré que celle-ci était influencée par l´activité de la maladie et les troubles psychiatriques [11].

En 2019, Fanouriakis *et al*. dans un groupe de travail sur la mise au point de la prise en charge du LES recommandent que le traitement des *NPSLE* soient ciblés en fonction de l´étiologie qu´elle soit thrombo-embolique soit inflammatoire, bien que ces 2 entités soient associées dans la plupart des cas [12]. L´augmentation du risque d´atteintes vasculaires chez le sujet noir à fort retentissement cérébral et son association à la prédisposition chez le sujet noir lupique, concourt à la gravité de la maladie et à un risque de développer un *NPSLE*. Doualla *et al*. en Afrique Centrale a montré une prédominance chez la jeune femme noire [13]. En Afrique de l´Ouest une étude a montré que la présence de manifestations cardio-vasculaires au cours de la maladie lupique augmentait le risque de déficit neurologique focal [14]. En Afrique subsaharienne, les données sur le LED sont rares, nous nous proposons d´étudier les *NPSLE* chez des patients consultant dans trois hôpitaux du Cameroun.

## Méthodes

**Cadre de l´étude:** nous avons mené une étude longitudinale rétrospective sur les dossiers médicaux patients lupiques suivis en rhumatologie durant la période allant de 2009 à 2019 au sein de 03 hôpitaux de référence du Cameroun: l´Hôpital Général de Douala (HGD), l´Hôpital Laquintinie de Douala (HLD) et l´Hôpital Central de Yaoundé (HCY).

**Contexte organisationnel:** L´HGD est une institution hospitalo-universitaire située au premier niveau de la pyramide sanitaire du Cameroun. L´unité de neurologie du service de médecine interne de l´HGD dispose de 4 neurologues, 2 secteurs d´hospitalisation, une unité de soins intensifs vasculaires, 1 secteur de consultations externes, 1 secteur d´explorations neurophysiologiques disposant de 02 appareils d´électroencéphalographie de marque Micromed et d´un électro-neuromyographe de marque Deltamed et 2 secteurs d´archives dont un hospitalier commun aux autres services de médecine et un service de consultation externe. L´unité de rhumatologie dispose de 2 rhumatologues, 2 secteurs d´hospitalisation, une unité de soins intensifs vasculaires, 1 secteur de consultations externes, 2 secteurs d´archives dont un hospitalier commun aux autres services de médecine et un service de consultation externe.

L´HLD est une formation hospitalo-universitaire située au deuxième niveau de la pyramide sanitaire du Cameroun. Le service de rhumatologie est réparti en deux secteurs: un secteur de consultation externe et un secteur d´hospitalisation localisé dans un bâtiment dans lequel on retrouve également le secteur d´hospitalisation du service d´hépato-gastro-entérologie. Elle dispose de 3 rhumatologues à temps plein. Le service de neurologie est réparti en deux secteurs: un secteur de consultation externe et un secteur d´hospitalisation localisé dans un bâtiment dans lequel on retrouve une unité de soins intensifs neurologiques et une unité de neurophysiologie. Elle dispose de 6 neurologues à temps plein.

L´HCY est une formation sanitaire hospitalo-universitaire. Le service de neurologie et de médecine physique comprend 10 salles d´hospitalisation, 3 salles d´infirmiers dont une dans laquelle sont rangés les dossiers des patients hospitalisés, une salle pour résidents, une salle pour étudiants, une salle d´archives commune et 4 neurologues. Le service de rhumatologie comprend 4 salles d´hospitalisations, 1 salle d´infirmiers, une salle pour résidents, une salle d´archives de dossiers des patients hospitalisés et des patients suivis en externe et 3 rhumatologues. Notre étude s´est déroulée sur 06 mois du 1^er^ janvier au 30 juin 2019 et concernait les patients hospitalisés ou reçus en consultation pour le LES pendant la période allant de 2009 à 2019.

**Participants:** notre population d´étude était constituée des dossiers des patients ayant comme diagnostic le LES et/ou suivis en externe ou hospitalisés dans les formations sanitaires choisies pour notre étude.

**Critères d´inclusion:** tous les dossiers des patients chez qui le diagnostic de LES a été posé selon les critères 1997 de *l´American College of Rheumatology (ACR)*, ainsi que les dossiers de patients présentant un ou plusieurs signes, symptômes et/ou pathologies faisant évoquer le diagnostic de neurolupus selon les critères *ACR* du *Neuropsychiatric Systemic Lupus Erythematosus (NPSLE)* de 1999.

**Critères d´exclusion:** les dossiers incomplets c´est à dire ne comportant pas les critères *ACR* nécessaires au diagnostic de LES étaient exclus. Les patients présentant un syndrome de chevauchement c´est-à-dire: ensemble de signes et symptômes retrouvés chez des patients remplissant des critères de plusieurs maladies systémiques étaient également exclus. La collecte de données s´est faite à l´aide des fiches techniques standardisées à partir des dossiers des patients hospitalisés et/ou suivis en consultations externes. Les données recueillies ont été analysées grâce au logiciel *Statistical Package for Social Science (SPSS)* version 20. Les variables qualitatives étaient exprimées en effectifs avec leurs pourcentages, tandis que les variables quantitatives étaient exprimées en moyennes et écart types. Une association entre variables qualitatives a été recherchée grâce au test de Fisher ou de chi-deux. Le test de student a été utilisé pour comparer les moyennes. L´estimateur de survie Kaplan Meir a été utilisé dans un premier temps pour comparer la probabilité de récidives des poussées au cours du suivi entre les patients lupiques avec ou sans atteinte neuropsychiatrique et dans un second temps pour comparer la probabilité d´avoir une activité lupique élevée au cours du suivi dans ces mêmes populations.

**Variables:** elles ont été étudiées:

**Les données sociodémographiques:** l´âge, le sexe, la région d´origine, le statut matrimonial, le niveau d´étude, l´âge au moment du diagnostic, le délai du diagnostic, la durée d´évolution du LES.

Le diagnostic de LES était retenu selon les critères *ACR* modifiés de 1997.

Les caractéristiques cliniques neuropsychiatriques étaient retenues selon les critères 1999 de *l´ACR*: méningite aseptique, syndrome de Guillain-Barré, atteinte cérébrovasculaire, atteinte du système nerveux autonome, syndrome démyélinisant, mononeuropathie, céphalées, myasthénie, convulsions, atteinte des paires des nerfs crâniens, état confusionnel aigu, plexopathie, myélite transverse, polyneuropathie, mouvements anormaux (chorée), dysfonction cognitive, troubles de l´humeur, troubles anxieux, psychose.

L´évaluation de l´activité lupique était mesurée par plusieurs items: le systemic lupus erythematosus disease activity index (SLEDAI) chez chaque patient qui a été interprété comme suit, pas d´activité (SLEDAI=0), activité légère (SLEDAI entre 1 et 5), activité moyenne (SLEDAI entre 6 et 10), activité élevée (SLEDAI entre 11 et 19), très haute activité (SLEDAI ≥20); les types de manifestations cliniques inaugurales du LES; le nombre de poussées; les durées entre la survenue des poussées.

Les manifestations d´atteintes cliniques et paracliniques des autres systèmes retenus d´après la littérature et pouvant survenir chez les malades lupiques ont également été rapportés.

**Source des données:** après obtention des autorisations de recherche des directions de structures hospitalières concernées et insertion dans les différents services, nous avons procédé au recensement année par année des différents patients enregistrés dans les registres d´hospitalisations et de consultations. La collecte de données après avoir testé notre questionnaire s´est faite par le biais des fiches techniques spécifiques aux services d´archives d´hospitalisation et de consultations externes de médecine interne des différents hôpitaux. La confidentialité des données a été respectée en attribuant un code spécifique à chaque fiche de collecte de données.

**Biais:** au cours de la collecte des données nous avons rencontré des difficultés dans la qualité d´entretien des dossiers et au report des informations dans lesdits dossiers.

**Taille de l´échantillon:** nous avons réalisé un échantillonage exhaustif non probabiliste, effectué dans les registre d´hospitalisations et de consultations externes.

**Variables quantitatives:** les variables quantitatives (âge, âge au diagnostic, durée d´évolution du LED, délai au diagnostic du LED) étaient exprimées en moyennes et écart types.

**Méthodes statistiques:** les données étaient entrées dans une base de données et analysées à l'aide du logiciel Statistical *Package for Social Sciences (SPSS)*, version 20.0. Les tableaux et les graphiques ont été mis en forme grâce aux logiciels Microsoft Word et Excel 2013. Notre population d´étude était subdivisée en (LES, *NPSLE* inauguraux et *NPSLE* non inauguraux). Les variables qualitatives étaient exprimées en effectifs avec leurs pourcentages. Une association entre variables qualitatives a été recherchée grâce au test de Fisher, de chi-deux. Le test de student a été utilisé pour comparer les moyennes. L´estimateur de survie Kaplan Meir a été utilisé dans un premier temps pour comparer la probabilité de récidives des poussées au cours du suivi dans les populations lupiques et neurolupiques et dans un second temps pour comparer la probabilité d´avoir une activité lupique élevée au cours du suivi dans ces mêmes populations.

**Considérations éthiques:** pour mener cette étude, nous avons obtenu une clairance éthique auprès du Comité d´éthique de l´Université de Douala (N 1794 CEI-Udo/04/2019/T) du 16 Avril 2019. Nous avons également obtenu des autorisations administratives de recherche des 3 hôpitaux concernés. La confidentialité des malades était préservée.

## Résultats

A la fin de notre étude, 108 dossiers de patients répondant aux critères de *l´ACR* 1997 ont été inclus. Les femmes représentaient 96,3% de la population d´étude et 58,8% étaient originaires de la région de l'Ouest Cameroun. L´âge moyen était de 40,2 ± 13,7 ans avec un minimum de 13 ans et un maximum de 75 ans. Au moment du diagnostic, l´âge moyen était de 27,2 ± 7,8 ans (Tableau 1).

**Tableau 1:** caractéristiques sociodémographiques des patients lupiques

Caractéristiques sociodémographiques	Effectifs	Pourcentage (%)
**Sexe**		
Féminin	104	96,3
Masculin	4	3,7
**Catégorie d'âge**		
≤ 20	3	2,8
21-30	31	28,7
31-40	22	25,0
41-50	27	20,4
51-60	15	13,9
60-70	8	7,4
˃70	2	1,9
**Région d'origine**		
Adamaoua	1	1,0
Centre	15	15,5
Est	1	1,0
Extrême-nord	3	3,1
Littoral	15	15,5
Nord	2	2,1
Nord-ouest	2	2,1
Ouest	57	58,8
Sud	1	1,0
Sud-ouest	0	0,0
**Statut marital**		
Célibataire	18	36,0
Divorcée	4	8,0
Mariée	25	50,0
Veuve	3	6,0
**Niveau d'étude**		
Non scolarisé	1	1,9
Primaire	19	36,5
Secondaire	6	11,5
Supérieure	26	50,0
**Secteur d'activité**		
Secteur formel	14	48, 3
Secteur informel	15	51,7

Les atteintes ostéo articulaire, dermatologique et immunoallergique étaient prépondérantes au sein de notre échantillon avec des fréquences respectives de 95,8%, 72,9% et 64,6%. La moitié des patients avaient une atteinte rénale (Tableau 2). La fréquence des *NPSLE* était de 55,5% (n=60). L´atteinte neuropsychiatrique était inaugurale chez 37,0% (n=40) des patients. Ces patients présentaient par ordre de fréquence le syndrome démyélinisant (n=11(27,5%)), des céphalées (n=11(27,5%)), la mononeuropathie (n=9, 22,5%) et les convulsions (n=4 (10,0%)) (Tableau 3). Au cours de la première année de suivi, 18,5% (n=20) des patients ont présenté des manifestions neuropsychiatriques. Les types de manifestations étaient comparables à ceux retrouvés lorsque le *NPSLE* était inaugural: syndrome démyélinisant (n=30 (27,8%)), les céphalées (n=23 (21,3%)) et la mononeuropathie (n=17(15,7%)).

**Tableau 2:** manifestations extra-neurologiques du LES

Variable	Effectif	Pourcentage %
Atteinte dermatologique	35	72,9
Alopécie	17	35,4
Rash malaire	23	47,9
Ulcération bucco pharyngée	4	8,3
Lupus discoïde	5	10,4
Photosensibilité	1	2,1
Macule hyperpigmenté	6	12,5
Atteinte ostéoarticulaire	46	95,8
Arthralgie	45	93,8
Arthrite	19	39,6
Myalgie	7	14,6
Myosite	2	4,2
Synovite	4	8,3
Atteinte rénale	24	50,0
Protéinurie	12	25,0
Hématurie	4	8,3
Cylindre urinaire	2	4,2
Leucocyturie	4	3,3
Œdème	12	25,0
Lésion biopsique	2	4,2
Atteinte cardiaque	6	12,5
Péricardite	5	10,4
Endocardite	1	2,1
Valvulopathie	1	2,1
Atteinte pleuropulmonaire	14	29,2
Douleur thoracique	3	6,3
Toux	3	6,3
Dyspnée	5	10,4
Pleurésie	9	18,8
Hypertension pulmonaire	1	2,1
Hémorragie intra alvéolaire	1	2,1
Atteinte immuno-hématologique	31	64,6
Adénopathie	3	6,3
Anémie	20	66,3
Leucopénie	10	20,8
Thrombopénie	3	6,3
Atteinte digestive	10	20,8
Dysphagie	3	6,3
Ascite	4	8,3
Atteinte immunobiologique	19	39,6

**Tableau 3:** types de manifestations neuropsychiatriques chez les patients lupiques

Variables, n=60	Effectif, n	Pourcentage,%
**Système nerveux central**		
Atteinte cérébro vasculaire	1	0,9
Psychose	1	0,9
Myélite transverse	2	1,9
Dysfonction cognitive	2	1,9
Confusion aigue	3	2,8
Méningite aseptique	4	3,8
Mouvement anormaux (chorée)	4	3,7
Trouble anxieux	5	4,6
Convulsion	7	6 ,5
Trouble de l'humeur	9	8,3
Céphalée	23	21,3
Syndrome démyélinisant	30	27,8
**Système nerveux périphérique**		
Polyneuropathie	1	0,9
Plexopathie	2	1,9
Dysautonomie	6	5,6
Neuropathie crânienne	8	7,4
Mononeuropathie	17	15,7

Les facteurs associés à une activité lupique élevée étaient l´arthralgie (p<0,001), la survenue d´un nouveau rash (p<0,001), l´antécédent d´un rash malaire (p=0,024), l´alopécie (p=0,024), la positivité du facteur anti-nucléaire (p=0,002), la présence d´une activité très élevée (p=0,011). Aucun facteur prédictif indépendant d´apparition de la *NPSLE* n´a été retrouvé (Tableau 4).

**Tableau 4:** analyse multi variée des facteurs prédictifs des manifestations neuro-psychiatriques du LED

Variables	Analyse uni varié		Analyse multi varié	
	OR non ajusté (IC 95%)	p value	OR ajusté (IC 95%)	p value
Arthralgie	188.000 (19.548-1808.024)	<0,001	0,456 (0,072-2,866)	0,402
Nouveau rash	47.000 (5.388-409.989)	<0,001	7,595 (0,844-68,312	0,070
Rash malaire	11.750 (1.222-113.001)	0.024	0,669 (0,276-1,621)	0,373
Alopécie	11.750 (1.222-113.001)	0.024	1.767 (0,681-4,582)	0,242
Pleurésie	8.294 (0.807-85.260)	0.073	0,468 (0,161-1,354)	0,161
Facteurs anti nucléaires	20.145 (2.233-181.700)	0.002	2,844 (0,252-32,081)	0,398
Activité lupique				
Activité légère	-----			
Activité moyenne	1.0909 (0.0606-19.6303)	0.740	0,615 (0,075-5,014)	0,649
Activité élevée	6.5455 (0.6765-63.3329)	0.088	0,538 (0,069-4,166)	0,552
Activité très élevée	12.000 (1.3028-110.5291)	0.011	1,529 (0,192-12,165)	0,688

**Figure 1:**
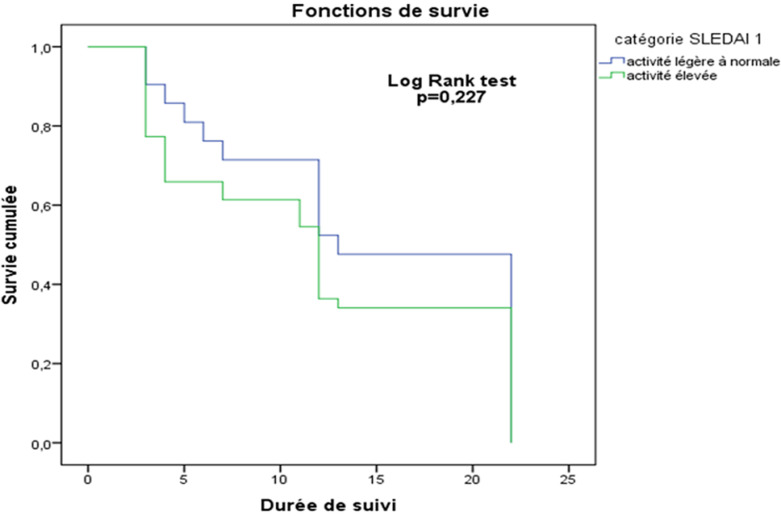
courbes de Kaplan Meier montrant la probabilité de survie des patients selon l’intensité de l’activité lupique

**Figure 2:**
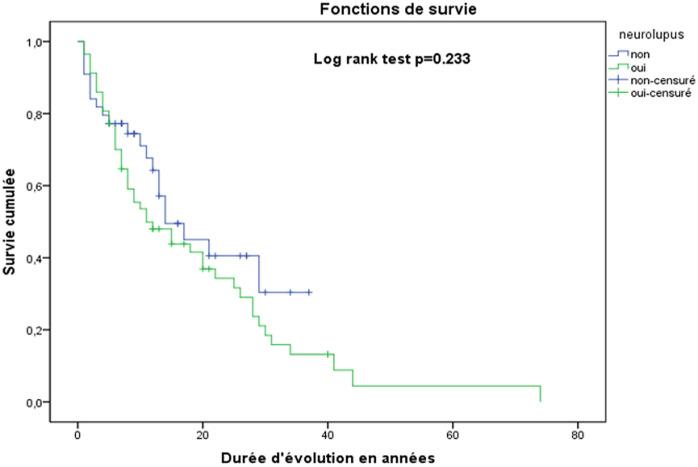
courbes de Kaplan Meier indiquant la probabilité de survenue des récidives des poussées lupiques

L´activité lupique élevée était observée chez 64,1% des patients (n = 37) mais il n´existait pas de différence significative entre les patients LES avec et sans atteintes neuropsychiatriques (log rank test p=0,227) (Figure 1). Après le diagnostic de LES, la moyenne de poussées lupiques au moment de l'étude était de 2,35 ± 1,61 fois avec des extrêmes de 2 pour la minima et 8 pour le maximum. La probabilité de récidive ne différait pas dans les deux populations (log rank test p=0,233) (Figure 2).

## Discussion

Le but de cette étude était de déterminer la fréquence des *NPSLE* au sein de trois hôpitaux de référence du Cameroun, de décrire les types de *NPSLE*, de rechercher les facteurs associés à leur survenue et de comparer l´impact de leur présence sur l´activité de la maladie lupique. Le LES est une maladie de cause inconnue fréquemment rencontré au sein des populations noires, notamment chez la jeune femme en âge de procréer. La littérature décrit une prévalence de neuf femmes atteintes pour un homme [15]. Dans notre population d´étude, la proportion des manifestations neuropsychiatriques était de 55,5%. Les manifestations neuropsychiatriques étaient inaugurales de la maladie chez 37,0% des cas au sein de la population lupique. Nos résultats sont similaires à ceux de Bertsias *et al*. en Grèce qui ont trouvé une proportion 50-60% de cas de *NPSLE* dont 40-50% étaient inauguraux de la maladie [16], et de Brey *et al*. menée à San Antonio avec une proportion de 80% de *NPSLE* [17]. Néanmoins de nombreuses études ont trouvé une proportion relativement faible. Xue Li *et al*. à Pékin ont trouvé une proportion de 6,4% [18].

Cette prépondérance de *NPSLE* s´explique par la sensibilité du système nerveux au mécanisme inflammatoire de la maladie lupique. Le système nerveux de ses fonctions informatives sensorielles, d´intégration et de réponse motrice est communément connecté à chaque système par voie autonome ou somatique. Ce qui justifierait son retentissement important au cours de cette maladie par mécanisme direct ou indirect. Doualla *et al*. ont trouvé une proportion de 8% dans la ville de Douala [13]. Zomalheto *et al*. au Benin et dans la sous-région d´Afrique Occidentale n´a rapporté aucun cas de neurolupus [19]. Les différences sociales et démographiques notamment les divergences raciales contribuent à la divergence de résultats avec les études menées par Xue Li à Pekin. Une divergence environnementale explique aussi cette différence compt-tenu du taux élevé de pollution dans nos villes. D´autre part la divergence de méthodologies utilisées justifie la différence entre les résultats de Doualla *et al*. par le fait qu´il s´agissait d´une étude mono centrique menée uniquement dans le service de médecine interne de l´HGD, ce qui aurait conduit à une faible taille de son échantillon soit 39 cas de LES. Dans l´étude menée par Zomalheto, l´absence de cas de *NPSLE* pourrait s´expliquer par le fait qu´il n´ait utilisé que la classification *ACR* de 1997 qui ne tient pas compte de toute la complexité d´atteinte neuropsychiatrique pouvant survenir au cours du LES tel que retrouvé dans la classification *NPSLE* de *l´ACR* de 1999.

Les types de manifestations neuropsychiatriques rencontrés à l´issue de notre étude étaient diverses. Seize des 19 manifestations neuro-psychiatriques ont été identifiées et au moins une de ces manifestations a été retrouvée chez 55,5% des patients. Les manifestations neuropsychiatriques étaient dominées par l´atteinte du système nerveux central avec un syndrome démyélinisant 27,8%, des céphalées 21,3%. L´atteinte du système nerveux périphérique était dominée par la mononeuropathie 15,7%. Ces résultats sont différents de ceux retrouvés dans la littérature. La prévalence du syndrome démyélinisant était de 3% dans l´étude de Hanly *et al*. de 2% dans l´étude Aniala *et al*. et nulle dans l´étude de Breys *et al*. Celle des céphalées était de 54% dans l´étude Aniala *et al*. de 57% dans l´étude Breys *et al*. Celle des mononeuropathies étaient de 8% dans l´étude de Breys *et al*. nulle dans celle d´Aniale et Hanly. Cette différence peut être due à l´influence des investigations paracliniques. L´atteinte démyélinisante et la mononeuropathie étaient retenues majoritairement sur la base d´éléments cliniques dans notre étude contrairement à l´étude menée par Aniala *et al*. où seules des neuropathies de preuve paraclinique étaient retenues comme critère d´atteinte nerveuse périphérique [20]. La proportion des convulsions de notre étude n´était pas similaire celle de l´étude de Breys *et al*. qui avait un nombre plus élevé d´hommes [17] pourtant la crise convulsive est de sévérité et de fréquence plus importantes chez les sujets de sexe masculin [21]. D´autre part il s´agissait d´une étude longitudinale dont la proportion du neurolupus était plus élevée que la nôtre.

**Facteurs associés à la survenue des *NPSLE*:** il existait dans notre échantillon une association significative de six variables sur la centaine de variables utilisée. Les facteurs associés à la présence de manifestations neuropsychiatriques retrouvées étaient les signes dermatologiques, les signes osteoarticulaires, la positivité des facteurs anti-nucléaires, et l´activité lupique élevée. Ceci peut être expliqué par notre contexte anthropologique. En effet, dans nos cultures Il faudrait que la maladie soit extériorisée ou douloureuse pour être considérée comme grave [22]. L´absence de spécialistes repartis dans toute les régions et l´accessibilité géographique et financière aux soins, retardent le diagnostic et la prise en charge. Nos résultats n´étaient pas en corrélation avec les littératures récentes. Une étude réalisée par Hanly *et al*. a montré une association (p<0,05) entre la survenue des *NPSLE* et une utilisation plus fréquente de corticostéroïdes et d´immunosuppresseur, de même qu´avec une baisse de la qualité de vie [23]. Une méta-analyse menée par Tay SH *et al*. a montré une association entre des sous unités du récepteur anti-N-méthyl-d-aspartate NR2A/B (anticorps anti-NR2A/B) et la survenue de *NPSLE* [24]. Ho RC *et al*. a montré une association entre des taux sériques élevés d´anticorps anti-cardiolipine (Acl), anticoagulant (LA), anti phospholipide (APL) et les *NPSLE* [25]. Cette divergence se justifierait par plusieurs arguments notamment le fait qu´il s´agissait de méta-analyse et qu´elles sont la résultante de plusieurs études menées sur le même sujet.

La probabilité de récidive des poussées, de même que celle d´une activité lupique élevée au cours de la durée de survie ne différent pas dans les deux populations. Ceci pourrait s´expliquer par l´influence indépendante d´autres facteurs favorisant une augmentation de récidive des poussées de façon proportionnelle au sein des différentes populations tel qu´une non observance au traitement, une culture médicale rétrograde, où la maladie est encore perçue comme ayant une origine surnaturelle, surtout lorsqu´elle est chronique [22].

L´absence de divergence entre la probabilité d´une activité lupique élevée au sein des populations avec et sans NP s´explique par l´utilisation dans notre étude d´un score d´activité lupique globale [26] (SLEDAI), dans lequel les atteintes NP sont regroupées en une minorité de critères comparés aux autres atteintes. D´autre part il existe d´autres atteintes de mauvais pronostic qui pourraient influencer l´activité de la maladie [2].

**Limites de l´étude:** les faiblesses de cette étude étaient le faible échantillon, et son caractère rétrospectif.

## Conclusion

Plus d´un patient sur deux atteint de LED présente une atteinte neuro-psychiatrique dont les plus fréquents sont le syndrome démyélinisant, les céphalées et la mononeuropathie. Cette atteinte est associée à une activité lupique très élevée entre patients avec ou sans atteinte neuro-psychiatrique. La probabilité de survenue d´une nouvelle poussée lupique ne différait pas des patients avec ou sans atteinte neuro-psychiatrique.

### Etat des connaissances sur le sujet

- L´atteinte neuropsychiatrique est possible au cours du LES avec des proportions variables d´un continent à l´autre;
- Par ailleurs, cette atteinte neuropsychiatrique est associée à la sévérité de l´activité de la maladie.

### Contribution de notre étude à la connaissance

- Notre étude permet de dire que plus d´un patient atteint de LES présente une atteinte neuropsychiatrique;
- L´activité lupique est élevée chez les patients lupiques avec atteinte neuropsychiatrique sans différence avec les patients lupiques sans atteinte neuropsychiatrique atteinte.

## Remerciements

Nous remercions le personnel des services de médecine interne et de rhumatologie de l´Hôpital Général de Douala, de l´Hôpital Laquintinie de Douala et de l´Hôpital Central de Douala pour leur contribution à l´aboutissement de ce travail.
